# Molecular prey identification in Central European piscivores

**DOI:** 10.1111/1755-0998.12436

**Published:** 2015-06-21

**Authors:** Bettina Thalinger, Johannes Oehm, Hannes Mayr, Armin Obwexer, Christiane Zeisler, Michael Traugott

**Affiliations:** ^1^ Institute of EcologyUniversity of InnsbruckTechnikerstraße 256020InnsbruckAustria

**Keywords:** *Alcedo atthis*, diagnostic fish primers, *Lutra lutra*, molecular scatology, *Phalacrocorax carbo sinensis*, piscivory

## Abstract

Diet analysis is an important aspect when investigating the ecology of fish‐eating animals and essential for assessing their functional role in food webs across aquatic and terrestrial ecosystems. The identification of fish remains in dietary samples, however, can be time‐consuming and unsatisfying using conventional morphological analysis of prey remains. Here, we present a two‐step multiplex PCR system, comprised of six assays, allowing for rapid, sensitive and specific detection of fish DNA in dietary samples. This approach encompasses 78 fish and lamprey species native to Central European freshwaters and enables the identification of 31 species, six genera, two families, two orders and two fish family clusters. All targeted taxa were successfully amplified from 25 template molecules, and each assay was specific when tested against a wide range of invertebrates and vertebrates inhabiting aquatic environments. The applicability of the multiplex PCR system was evaluated in a feeding trial, wherein it outperformed morphological prey analysis regarding species‐specific prey identification in faeces of Eurasian otters. Additionally, a wide spectrum of fish species was detected in field‐collected faecal samples and regurgitated pellets of Common Kingfishers and Great Cormorants, demonstrating the broad applicability of the approach. In conclusion, this multiplex PCR system provides an efficient, easy to use and cost‐effective tool for assessing the trophic ecology of piscivores in Central Europe. Furthermore, the multiplex PCRs and the primers described therein will be applicable wherever DNA of the targeted fish species needs to be detected at high sensitivity and specificity.

## Introduction

The borders between aquatic and terrestrial ecosystems exhibit extraordinary diversity, productivity and manifold interactions between organisms (Polis *et al*. 1997). Movements of nutrients, prey and predators influence the structure and productivity of both the donor and the recipient system (Burdon & Harding 2007). In this regard, piscivores play a pivotal role as consumers and vectors (Polis *et al*. 2004; Ellis *et al*. 2006). They induce trophic cascades or general top‐down effects (Schmitz *et al*. 2010) and thus alter aquatic and terrestrial ecosystems at individual, population, community and ecosystem level (Polis *et al*. 2004; Baxter *et al*. 2005; Knight *et al*. 2005).

Piscivores such as the Common Kingfisher (*Alcedo atthis,* further on ‘kingfisher’), Bald Eagle (*Haliaeetus leucocephalus*) and Eurasian otter (*Lutra lutra,* further on ‘otter’) serve as ecosystem indicators and even flagship species for nature conservation (Entwistle & Dunstone 2000; Clucas *et al*. 2008). They provide key supporting and regulating ecosystem services (Green & Elmberg 2014) and indicate protection worthy, high levels of biodiversity (Sergio *et al*. 2006). In Europe, such piscivores and the freshwater ecosystems they inhabit are protected under the Council Directive 92/43/EEC and the Directive 2009/147/EC of the European Union (Council of the European Union 1992; European Parliament & Council of the European Union 2009). In contrast, piscivores with high population densities such as cormorants, herons or pinnipeds have long been perceived as food competitors by humans (Duffy 1995; Gosch *et al*. 2014) resulting in shootings, culling and repellent measures (Boudewijn & Dirksen 1999; Bowen & Lidgard 2013). Any management of piscivores aiming at either protecting or regulating these animals depends on a sound understanding of their feeding ecology. Consequently, a low‐cost method, enabling the identification of prey species at high taxonomic resolution, is a sorely needed tool to analyse non‐invasively gained dietary samples and shape conservation and management efforts along European freshwaters.

Dietary studies on piscivores in the traditional sense involve stomach content analysis of killed specim‐ens (Labansen *et al*. 2007; Bostrom *et al*. 2012), stomach flushing (Hull 1999; Alonso *et al*. 2014), direct observation (Gonzalez‐Solis *et al*. 1997), as well as morphological hard part analysis of indigestible prey remains in regurgitated pellets (Gonzalez‐Solis *et al*. 1997; Dias *et al*. 2012) and faeces (Jedrzejewska *et al*. 2001; Sinclair & Zeppelin 2002). The morphological analysis of prey remains found in pellets and faeces permits conclusions about the number and size of the consumed fish prey (Mariano‐Jelicich & Favero 2006; Gagliardi *et al*. 2007). There are, however, numerous disadvantages to these approaches such as the limited applicability of stomach flushing, digestion bias due to prey size and species, and often species‐specific identification is not possible (reviewed and discussed in Barrett *et al*. 2007; Bowen & Iverson 2012). DNA‐based prey identification can overcome many of these limitations, and a variety of molecular methods have been developed over the past 15 years enabling the investigation of feeding interactions across trophic levels and ecosystems at unprecedented resolution (King *et al*. 2008; Pompanon *et al*. 2012; Traugott *et al*. 2013; Symondson & Harwood 2014). Hence, even morphologically unidentifiable prey remains can be exploited to track trophic links (Casper *et al*. 2007; Bowen & Iverson 2012). Molecular techniques have also been used to assess the diet of piscivores with a focus on marine predators such as pinnipeds (Deagle *et al*. 2009; Marshall *et al*. 2010), squids (Deagle *et al*. 2005) and seabirds (Bowser *et al*. 2013; Jarman *et al*. 2013), but to this point, they have only been scarcely applied in freshwater ecosystems (e.g. Bradford *et al*. 2014; Brandl *et al*. 2014).

Whilst sequence‐based methods such as next‐generation sequencing (further on ‘NGS’) provide information on the prey range at high taxonomic resolution, they are time‐consuming and expensive, especially when dealing with high sample numbers (Pompanon *et al*. 2012). Diagnostic multiplex PCR provides a valuable alternative to sequence‐based approaches when a defined set of prey organisms is to be detected: multiplexing of taxon‐specific primers allows the identification of several prey taxa within one reaction, based on differences in amplicon size (Harper *et al*. 2005; Sint *et al*. 2012). Depending on the information needed, the taxonomic level of prey identification can be selected and through balancing primer concentrations, equal sensitivity can be reached across the targeted taxa (Sint *et al*. 2012). Diagnostic multiplex PCR works best for investigating trophic interactions in an environment with a limited and predictable number of prey species (Symondson & Harwood 2014), as for example, fish in Central European freshwaters. Nevertheless, the number of fish species in this environment exceeds the number of targets possible in one multiplex PCR. For such situations, we propose a novel two‐step approach where targets are initially identified at a high taxonomic level and species‐specific identification is carried out in the respective follow‐up PCRs (Caballero *et al*. 2012).

The aims of the present study were threefold: (I) to develop a two‐step multiplex PCR system for efficient, easy and sensitivity‐balanced identification of DNA from Central European freshwater fish in dietary samples and thus providing an alternative to the more time‐intensive and expensive sequence‐based methods, (II) to compare the performance of the new system with morphological scat analysis in a feeding trial with otters and (III) to apply the molecular detection system to field‐collected faecal samples of kingfishers and cormorants as well as to cormorant pellets.

## Materials and methods

### Origin of reference samples for DNA extraction and sequence generation

Between 2011 and 2013, tissue samples of 78 fish and lamprey species occurring in Central Europe (Rhine and Danube catchment) were collected. Species inhabiting coastal and brackish waters, except the Atlantic salmon, were not included. For very rare or endangered species, tissue samples or DNA extracts were provided by museums and scientific collections (Table S1, not included red‐listed species see Appendix S1, Supporting information). Additionally, nontarget organisms occurring in lotic and lentic freshwaters including gastropods, amphibians, reptiles and arthropods were collected (Table S2, Supporting information). Tissue samples of piscivorous mammals and birds were provided by the zoological collection of the Tiroler Landesmuseen (Table S2, Supporting information). All samples were DNA extracted using the DNeasy blood and tissue kit (QIAGEN, Hilden, Germany) following the manufacturer's instructions.

Fragments of the mitochondrial 16S rRNA (16S) gene and cytochrome *c* oxidase subunit I (COI) gene were amplified for all target species (Table S1, Supporting information) using the forward primer 16Sar plus the reverse primer 16Sbr for 16S (Gleason & Burton 2012) and the forward primer FishF1 plus the reverse primer FishR1 for COI (Ward *et al*. 2005), respectively. Purified PCR products were sequenced by Eurofins MWG Operon (Munich, Germany); the sequences were edited using BioEdit (Hall 1999) and their identity checked by blast (NCBI website http://blast.ncbi.nlm.nih.gov/Blast.cgi). Thereafter, 16S and COI consensus sequences were created for each species, using the sequences derived from the DNA extracts in combination with sequences already available on GenBank (NCBI) or BOLD (http://www.boldsystems.org/) and aligned across species in BioEdit.

### Primer design and multiplex PCR development

Using Primer Premier 5 (PREMIER Biosoft International, Palo Alto, CA, USA), 82 primers with melting temperatures as close as possible to 60 °C were designed and arranged in six multiplex PCR assays. Within each assay, primer pairs were employed whose amplicons exhibited at least a 20‐bp difference in length to ensure proper separation in electrophoresis (see Appendix S2 for exception, Supporting information).

Initially, the functioning of all primer pairs was tested in singleplex PCRs. Thereafter, they were combined to multiplex PCR assays whose optimal annealing temperatures were determined by gradient PCR and the primer concentrations were adjusted to balance the sensitivity across primer pairs within each assay using standardized DNA templates (Sint *et al*. 2012). These target fish species DNA templates were generated from PCR products also used for sequencing. All reactions were based on the Multiplex PCR Kit (QIAGEN) using additional bovine serum albumin (BSA) to prevent PCR inhibition (Juen & Traugott 2006) and tetramethylammonium chloride (TMAC) to enhance specificity (Chevet *et al*. 1995). Standardized DNA templates of target species (Table S1, Supporting information) were used to determine the sensitivity of each primer pair within the six multiplex PCRs in the presence of ~300 ng cormorant DNA to simulate field‐collected dietary samples. An extensive specificity testing was carried out by applying the multiplex PCR assays to 632 DNA extracts of the targeted fish species (Table S1, Supporting information) and 113 nontarget samples representing 61 taxa (Table S2, Supporting information) to rule out false‐positive amplifications. Any additional PCR amplicons produced by DNA extracts during specificity testing were sequenced and confirmed to be contaminations in the extracts. These were subsequently replaced by uncontaminated DNA extracts generated from fish muscle tissue.

qiaxcel, an automatic capillary electrophoresis system with the corresponding software qiaxcel biocalculator version 3.2 (Method AL320; QIAGEN), was used for PCR product separation and analysis. All DNA fragments of the expected fragment size producing a signal strength above ≥0.07 relative fluorescent units (RFU) were deemed positive. Finally, *in silico* PCRs were carried out for all multiplex PCR assays with CLC Main Workbench 7 (CLC bio, Aarhus, Denmark) using the ‘Find Binding Sites and Create Fragments’ tool. The 16S and COI sequences of European freshwater Mollusca, Ephemeroptera, Plecoptera, Trichoptera, Zygoptera and Chironomidae available online at GenBank were used as a basis for these calculations (see Table S4 for detailed settings, Supporting information).

### Multiplex PCR evaluation via feeding trial and field‐collected samples

In November 2013, a feeding trial with three Eurasian otters (*Lutra lutra*) was conducted at the Alpenzoo (Innsbruck, Austria). Otters were housed in a 142 m^2^ enclosure including a large pond (⅔ of enclosure surface), rocks and tree trunks to rest on, and a holt accessible from underwater. The otters were a family comprised of mother, father and son and at that time eight, six and one year old, respectively. Three days prior to the trial, they were fed a fish‐free diet consisting of day‐old chicks, chinchillas and guinea pigs (~2000 g per day). Thereafter, four different fish species were offered, one per evening, starting with 2500 g rainbow trout (*Oncorhynchus mykiss*) followed by 2025 g roach (*Rutilus rutilus*), 917 g perch (*Perca fluviatilis*) and 552 g whitefish (*Coregonus* spp.). All fish had been gilled and thoroughly rinsed under flowing water prior to the trial. The following 3 days the otters’ diet was kept fish‐free again and consisted of day‐old chicks and cattle heart (~2000 g per day). Five faecal samples (spraints) were collected each evening starting 1 day before rainbow trout was provided and ending 3 days after whitefish was offered. All spraints and field‐collected samples were individually collected in plastic bags or reaction tubes using gloves, frozen in cooling boxes in the zoo or field and stored at −80 °C until DNA extraction.

Concerning field‐collected dietary samples, on 11 and 12 June 2011 seven kingfisher faeces were collected on the riverbanks of Danube, March and Thaya in Germany and Austria after observing the birds defecate (see Table S3 for locations, Supporting information). Forty‐five faecal samples of cormorants were collected on 20 December 2012 under roosting trees along the Chiemsee shoreline (N47.85964, E12.51174, Germany), and 45 regurgitated cormorant pellets were collected on 1 February 2013 on a small island in the Chiemsee (N47.869092, E12.416847, Germany).

### Processing of scat samples and pellets

All zoo‐ and field‐collected samples were lysed with a mixture of TES‐buffer (0.1 m TRIS, 10 mm EDTA, 2% sodium dodecyl sulphate; pH 8) and proteinase K (20 mg/ml) in a ratio of 190:1. The amount of lysis buffer added to the sample depended on its size: 6 ml for small (5 to 10 cm³), 8 ml for large (10 to 20 cm³) otter spraints and 300 *μ*l for kingfisher faeces. Cormorant pellets were separated into three size classes with 3 ml for small, 5 ml for medium and 8 ml for large samples. Likewise, 300 *μ*l, 500 *μ*l and 3 ml of lysis buffer were used for small, medium and large cormorant faeces, respectively. After adding the lysis buffer, all samples were vortexed and incubated overnight at 56 °C in a rocking platform.

DNA extraction was carried out with the BioSprint 96 instrument (QIAGEN) using the BioSprint 96 DNA blood Kit (QIAGEN) in accordance with the manufacturer's instructions. Each otter spraint lysate was extracted three times to maximize the chances of DNA detection (Oehm *et al*. 2011). Per BioSprint run 92 lysates and four blank extraction controls were processed. Controls contained TES‐buffer instead of lysate and were checked with the family‐specific (FishTax) multiplex PCR assay (see Results section) for cross‐contamination potentially occurring during the DNA extraction process. All of them resulted negative. The obtained DNA extracts were subsequently analysed with the two‐step multiplex PCR system (see Results section), and samples testing negative in the FishTax multiplex PCR (see Results section) were spiked with ~50 ng perch DNA to test for PCR inhibition. For some spraints, prey identification was only possible at order level. These PCR amplicons produced by order‐specific primer pairs were sequenced to resolve fish species identity via sequencing.

Prior to the morphological analysis, the dissolved otter spraints were strained and rinsed with distilled water. Identifiable fish hard parts such as lenses, scales, vertebras, chewing pads and jaws were sorted out and identified using the keys of Knolleisen (1996) and Čech (2006) as well as using fish bone reference collections provided by Dr. Werner Suter (Swiss Federal Research Institute, Birmensdorf, Switzerland), Dr. Josef Trauttmansdorff (Otto‐König Institute, Stockerau, Austria) and the Bavarian State Collection of Zoology (Munich, Germany). A chi‐squared test was calculated to check for significant differences between the molecular and the morphological approach regarding species‐specific prey detection in the otter spraints using MS Excel 2010.

## Results

### Multiplex PCR system

**Figure 1:**
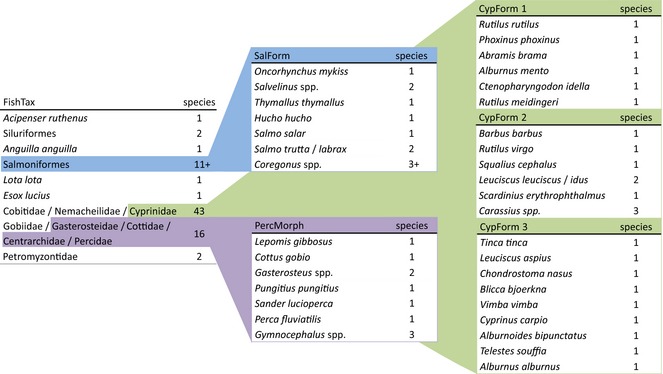
The two‐step multiplex PCR system comprising six assays (FishTax, SalForm, PercMorph, CypForm 1–3) to identify fish DNA in dietary samples, depicting the assays and the identity and number of the target taxa. Coloured areas indicate which target groups from the FishTax assay are subjected to further identification.

**Figure 2:**
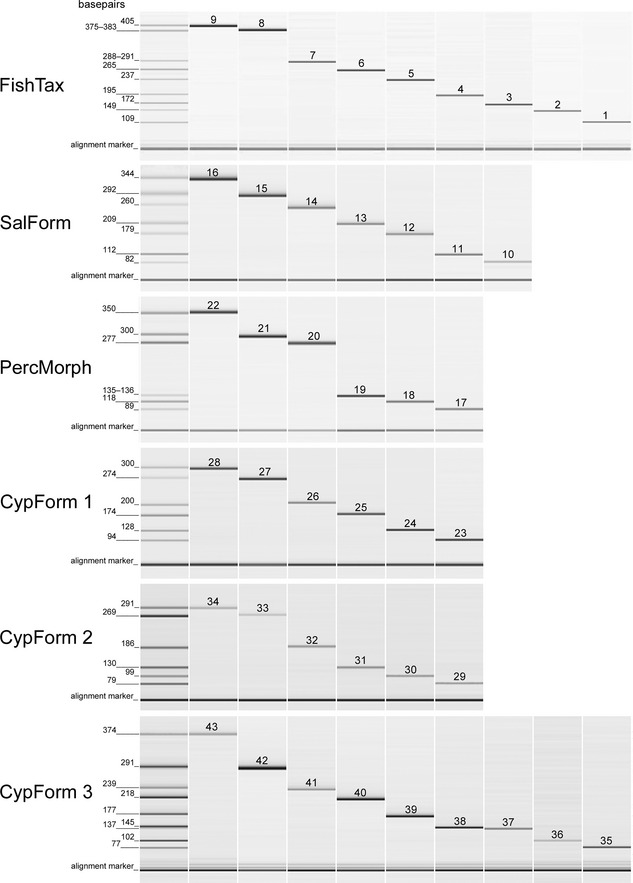
qiaxcel gel view of amplicons generated by the diagnostic multiplex PCR assays. The leftmost lane shows a mixture of all targeted taxa per reaction with equal target DNA concentrations and the amplicon lengths in base pairs. The single bands displayed in the other lanes were generated with ~150 double strands of target template DNA in the presence of ~300 ng nontarget DNA (*Phalacrocorax carbo sinensis*). FishTax: 1: *Acipenser ruthenus*, 2: Siluriformes, 3: *Anguilla anguilla*, 4: Salmoniformes, 5: *Lota lota*, 6: *Esox lucius*, 7: Cobitidae/Nemacheilidae/Cyprinidae, 8: Gobiidae/Gasterosteidae/Cottidae/Centrarchidae/Percidae, 9: Petromyzontidae. SalForm: 10: *Oncorhynchus mykiss*, 11: *Salvelinus* spp., 12: *Thymallus thymallus*, 13: *Hucho hucho*, 14: *Salmo salar*, 15: *Salmo trutta*/*labrax*, 16: *Coregonus* spp. PercMorph: 17: *Lepomis gibbosus*, 18: *Cottus gobio*, 19: *Gasterosteus* spp./*Pungitius pungitius*, 20: *Sander lucioperca*, 21: *Perca fluviatilis*, 22: *Gymnocephalus* spp. CypForm 1: 23: *Rutilus rutilus*, 24: *Phoxinus phoxinus*, 25: *Abramis brama*, 26: *Alburnus mento*, 27: *Ctenopharyngodon idella*, 28: *Rutilus meidingeri*. CypForm 2: 29: *Barbus barbus*, 30: *Rutilus virgo*, 31: *Squalius cephalus*, 32: *Leuciscus leuciscus*/*idus*, 33: *Scardinius eryhthrophthalmus*, 34: *Carassius* spp. CypForm 3: 35: *Tinca tinca*, 36: *Leuciscus aspius*, 37: *Chondrostoma nasus*, 38: *Blicca bjoerkna*, 39: *Vimba vimba*, 40: *Cyprinus carpio*, 41: *Alburnoides bipunctatus*, 42: *Telestes souffia*, 43: *Alburnus alburnus*.

For the design of the multiplex PCR system, 311 fish DNA sequences encompassing 78 species were generated and the highest quality sequences uploaded to GenBank (Accession nos in Table S1, Supporting information). This includes 20 and four species with not so far public 16S and COI sequences (GenBank, as of 10 February 2015), respectively (Table S1, Supporting information). Based on these and online available sequence information, six multiplex PCRs were set up for identi‐fication of 31 fish species, six genera, two families, two orders and two fish family clusters using primer pairs amplifying DNA fragments between 77 bp and 405 bp (Figs 1 and 2, Table 1). In the first, family‐specific multiplex PCR assay (‘FishTax’) each of the 78 fish and lamprey species is assigned to one of nine target groups. These include one fish family, two orders and two family clusters resulting from DNA sequence dissimilarities between these target groups. The target groups are Petromyzontidae, Siluriformes, Salmoniformes, Cobitidae/Nemacheilidae/Cyprinidae (further on ‘Cypriniformes’; lowest shared taxonomic level) and Gobiidae/Gasterosteidae/Cottidae/Centrarchidae/Percidae (further on ‘Percomorphaceae’; lowest shared taxonomic level). Four fish species, which are genetically separated from all other taxa examined here, are targeted species specifically in the FishTax assay, namely sturgeon (*Acipenser ruthenus*), European eel (*Anguilla anguilla*), burbot (*Lota lota*) and pike (*Esox lucius*). Samples testing positive with the group‐specific primers in the FishTax assay are to be subjected to the respective second‐step multiplex PCR(s). These five follow‐up assays for species‐specific identification are ‘SalForm’, ’PercMorph’, ‘CypForm 1’, ‘CypForm 2’ and ‘CypForm 3’ (Fig. 1). The SalForm assay identifies species within the Salmoniformes (Fig. 1), except *Coregonus* and *Salvelinus* for which identification is limited to genus level, as well as the species combination of *Salmo trutta* and *Salmo labrax*. The ‘PercMorph’ multiplex PCR identifies four species, one genus and one family within the Percomorphaceae (Fig. 1); sticklebacks (Gasterosteidae) are in practice displayed as one 135‐ to 136‐bp diagnostic band, as amplicon sizes of *Pungitius pungitius* and *Gasterosteus* spp. differ by only 1 bp. For the species‐rich Cypriniformes, three assays (CypForm 1–3) were set up, identifying 19 species and two genera (Fig. 1).

**Table 1:** A summary of the multiplex PCR assays: the sensitivity of each multiplex PCR in DNA double strands (ds) necessary to reliably detect a target taxon in a sample with mixed target and nontarget DNA is provided. Target taxa, primer names and sequences, genes targeted by the primers and their corresponding amplicon sizes, primer concentrations in PCR and the number of target species per primer pair are specified too

Assay name	Sensitivity (DNA ds)	Target taxa	Primer name	Primer sequence (5′–3′)	Target gene	Fragment size (bp)	Primer concentration in PCR (*μ* m)	Number of target species
FishTax	17	*Acipenser ruthenus*	Gen‐mix‐S628	AATGAAGACCTGTATGAATGGCAT	16S	109	1.2	1
			Aci‐rut‐A633	CTTCTCGTCTTATGGGGTTATGCT	16S		0.2	
		Siluriformes	Sil‐for‐S629	CGCCTCCTGCAAAAATCAAY	16S	149	0.2	2
			Sil‐for‐A637	AGACAGTTAAGCCCTCGTTCCA	16S		0.2	
		*Anguilla anguilla*	Gen‐mix‐S628	AATGAAGACCTGTATGAATGGCAT	16S	172	a	1
			Ang‐ang‐A638	TGTTCCTTTTGGTTGGTTTGGT	16S		0.15	
		Salmoniformes	Gen‐mix‐S628	AATGAAGACCTGTATGAATGGCAT	16S	195	a	11+
			Sal‐for‐A631	CATAKGGGCTAGGGGTCACTG	16S		0.4	
		*Lota lota*	Gen‐mix‐S628	AATGAAGACCTGTATGAATGGCAT	16S	237	a	1
			Lot‐lot‐A635	CCACATGGGGGTTGTGTTTTA	16S		0.3	
		*Esox lucius*	Gen‐mix‐S628	AATGAAGACCTGTATGAATGGCAT	16S	265	a	1
			Eso‐luc‐A636	CGTGGTTATAAGGAGGTTTTCCTT	16S		0.2	
		Cobitidae, Nemacheilidae, Cyprinidae	Cyp‐cgo‐S627	GAGGTCCAGCCTGCCCA	16S	288–291	0.7	43
			Cyp‐cgo‐A630	CGCCCCAACCGAAGGTAA	16S		0.7	
		Gobiidae, Gasterosteidae, Cottidae, Centrarchidae, Percidae	Gen‐mix‐S628	AATGAAGACCTGTATGAATGGCAT	16S	375–383	a	16
			Per‐ngc‐A634	CCTTGTCGATRTGRGCTCTAAA	16S		0.3	
		Petromyzontidae	Pet‐myz‐S630	ATCGCCTATTGGAGGCAAGA	16S	405	0.5	2
			Pet‐myz‐A639	GGGGTAACTTGTTTCGTTAGGCA	16S		0.5	
SalForm	22	*Oncorhynchus mykiss*	Onc‐myk‐S655	TCTCCCTTCATTTAGCTGGAATC	COI	82	0.15	1
			Onc‐myk‐A655	GCTGGAGGTTTTATGTTAATAATGGTC	COI		0.15	
		*Salvelinus* spp.	Sal‐vel‐S651	ATAGTCGGCACCGCCCTT	COI	112	0.15	2
			Sal‐vel‐A651	TAACGAAGGCATGGGCTGTT	COI		0.15	
		*Thymallus thymallus*	Thy‐thy‐S653	ATCAAATTTATAATGTGATCGTCACG	COI	179	0.15	1
			Thy‐thy‐A653	AAGAAAGGACGGGGGAAGC	COI		0.15	
		*Hucho hucho*	Huc‐huc‐S650	TGATTTAACTATCTTCTCTCTCCACTTG	COI	209	0.15	1
			Huc‐huc‐A650	GGTCTGTAAGTAGTATAGTGATGCCC	COI		0.15	
		*Salmo salar*	Sal‐sal‐S656	TGGCGCCCTTCTGGGA	COI	260	0.40	1
			Sal‐sal‐A656	GGGGGTAGACTGTTCATCCG	COI		0.40	
		*Salmo trutta*/*labrax*	Sal‐tru‐S654	CTTTGGAAACTGATTAATCCCTCTC	COI	292	0.70	2
			Sal‐tru‐A654	TGGGGGTTTTATGTTAATAATGGTC	COI		0.70	
		*Coregonus* spp.	Cor‐spp‐S652	GATCAGATTTATAATGTAATCGTCACG	COI	344	0.50	3+
			Cor‐spp‐A652	ATAAAATTAACGGCCCCCAAG	COI		0.50	
PercMorph	25	*Lepomis gibbosus*	Lep‐gib‐S633	GGAGCTTTAGACGCCTGAGTAAG	16S	89	0.48	1
			Lep‐gib‐A642	CCCCAACCAAAGACATGGA	16S		0.48	
		*Cottus gobio Cottus gobio*	Cot‐gob‐S632	GAATAAAGGACTAAACCAAGTGGG	16S	118	0.48	1
			Cot‐gob‐A641	GCTGTAGCTCTCAGTTGTAGGAAAA	16S		0.48	
		*Gasterosteus* spp.	Gas‐ter‐S631	ACAAGATGGAACCCACCCTG	16S	136	0.24	2
			Gas‐ter‐A649	GATCTTTTTGGTCAGAAATTCTGTTTA	16S		0.4	
		*Pungitius pungitius*	Pun‐pun‐S640	CCAAATGGAACCCACCCTG	16S	135	0.5	1
			Gas‐ter‐A649	GATCTTTTTGGTCAGAAATTCTGTTTA	16S		b	
		*Sander lucioperca*	San‐luc‐S658	CTCCTTGCTTCCTCAGGGGTA	COI	277	0.16	1
			San‐luc‐A658	CGGCAAGTACGGGGAGC	COI		0.16	
		*Perca fluviatilis*	Per‐flu‐S671	GTACCGGGTGAACTGTATATCCG	COI	300	0.16	1
			Per‐flu‐A671	CAGGGTCAAAGAAAGTTGTGTTC	COI		0.16	
		*Gymnocephalus* spp.	Gym‐spp‐S677	CTCCTTGCTTCCTCAGGAGTA	COI	350	0.4	3
			Gym‐spp‐A657	AATGTTGGTAGAGGATGGGATCR	COI		0.4	
CypForm 1	13	*Rutilus rutilus*	Rut‐rut‐S665	TTCYGGTGTTGAGGCCGGT	COI	94	0.25	1
			Rut‐rut‐A665	TGTTAAATCTACTGATGCCCCG	COI		0.25	
		*Phoxinus phoxinus*	Pho‐Pho‐S639	CGTGCAGAAGCGGATATAAATAC	16S	128	0.175	1
			Pho‐Pho‐A648	CCAACCGAAGGTAAAGTCTTATTG	16S		0.175	
		*Abramis brama*	Abr‐bra‐S638	GGAGCTTAAGGTACAAAATTTAACCAT	16S	174	0.15	1
			Abr‐bra‐A647	CAGATGTTCTGCGGCTTATAGG	16S		0.15	
		*Alburnus mento*	Alb‐men‐S662	TTTCTGACTCCTTCCGCCG	COI	200	0.6	1
			Alb‐men‐A662	TGGTGGTAATGAAGTTGACTGCA	COI		0.6	
		*Ctenopharyngodon idella*	Cte‐ide‐S635	CGCCTCCTGCAATCAAACTC	16S	274	0.4	1
			Cte‐ide‐A644	CTTTTTATTGAGTTGCTTAACGTGA	16S		0.4	
		*Rutilus meidingeri*	Rut‐mei‐S666	CTACCCCCATCATTCCTATTATTGT	COI	300	0.2	1
			Rut‐mei‐A666	GGCAGCTAGCACTGGTAGTGAC	COI		0.2	
CypForm 2	25	*Barbus barbus*	Bar‐bar‐S637	CGTGCAGAAGCGGGTATAATAT	16S	79	0.4	1
			Bar‐bar‐A646	TTGCTTGACGTGGTTGATCTTTA	16S		0.4	
		*Rutilus virgo*	Rut‐vir‐S667	CCTACCCCCATCATTCCTATTATTAC	COI	99	0.4	1
			Rut‐vir‐A667	GCGAGGTTGCCTGCAAGC	COI		0.4	
		*Squalius cephalus*	Squ‐cep‐S669	CAGTATACCCACCGCTTGCG	COI	130	0.2	1
			Squ‐cep‐A669	TTAATAATTGTGGTAATGAAGTTGACC	COI		0.2	
		*Leuciscus leuciscus*/*idus*	Leu‐lid‐S663	CATCTCCCAGTATCAAACACCG	COI	186	0.2	2
			Leu‐lid‐A663	AATCAGAATAAGTGTTGGTACAGGATC	COI		0.2	
		*Scardinius eryhtrophthalmus*	Sca‐ery‐S668	GAGTTTCTGACTTCTCCCTCCG	COI	269	0.1	1
			Sca‐ery‐A668	CCAGTACGGCTCATACAAACAGC	COI		0.1	
		*Carassius* spp.	Car‐ass‐S659	GAGCTGGCACCGGATGG	COI	291	0.25	3
			Car‐ass‐A659	TGGTGTTAAGATTTCGATCTGTTAAA	COI		0.25	
CypForm 3 *Chondrostoma nasus*	17	*Tinca tinca*	Tin‐tin‐S636	GTACAAAATTCAACCACGTCAAGA	16S	77	0.4	1
			Tin‐tin‐A691	CCAACCGAAGGTAAAAGTTCATAA	16S		0.4	
		*Leuciscus aspius*	Leu‐asp‐S641	CACGTTAAACGACTCCGCAC	16S	102	0.2	1
			Leu‐asp‐A692	CCAATCCACTCGGAGGCTC	16S		0.2	
		*Chondrostoma nasus*	Cho‐nas‐S678	CTTCTACCCCCCTCATTCCTCT	COI	137	0.4	1
			Cho‐nas‐A678	GAGAAGATTGTTAAATCTACTGATGCA	COI		0.4	
		*Blicca bjoerkna*	Bli‐bjo‐S675	GGTCACTTTTAGGCGATGACCAG	COI	145	0.15	1
			Bli‐bjo‐A676	GCCATATCAGGCGCACCG	COI		0.15	
		*Vimba vimba*	Vim‐vim‐S676	AATCTCGCCCATGCTGGC	COI	177	0.4	1
			Vim‐vim‐A677	GACGGCTGTTACTAGTACGGCC	COI		0.7	
		*Cyprinus carpio*	Cyp‐car‐S660	CCCACCTCTTGCAGGGAACT	COI	218	0.5	1
			Cyp‐car‐A660	AAACAGGTAATGATAGAAGGAGCAAT	COI		0.5	
		*Alburnoides bipunctatus*	Alb‐bip‐S673	TGACTACTACCTCCATCATTTTTGC	COI	239	0.2	1
			Alb‐bip‐A674	GGTGTTTGATACTGGGAGATAGCC	COI		0.2	
		*Telestes souffia*	Tel‐sou‐S642	CATCGCCTCCTGCAACTAATC	16S	291	0.2	1
			Tel‐sou‐A693	CACCACTAAGTTCGTGCTTTCTATC	16S		0.2	
		*Alburnus alburnus*	Alb‐alb‐S672	CACGAATAAATAACATGAGTTTCTGG	COI	374	0.15	1
			Alb‐alb‐A673	AAGAATGTGGTATTAAGATTACGATCC	COI		0.1	

a Total concentration: 1.2 *μ* m; equals 6 × 0.2 *μ* m.

b Total concentration: 0.4 *μ* m; equals 2 × 0.2 *μ* m.

The 10 *μ*l PCRs were performed using the Multiplex PCR Kit (QIAGEN) including 3.2 *μ*l of DNA extract except for the FishTax assay, which contained 1.5 *μ*l. One‐time reaction mix, 5 *μ*g BSA, 30 mm TMAC, primers in respective concentrations (Table 1) and PCR‐grade water (FishTax assay only) were used in each reaction. The optimized thermocycling conditions were 15 min at 95 °C, 35 cycles of 30 s at 94 °C, 90 s at 64 °C (FishTax, SalForm, PercMorph, CypForm 2) or 66 °C (CypForm 1, CypForm 3), 1 min at 72 °C and 10 min at 72 °C once.

Within the multiplex PCRs described above, each primer was specific for its target taxon as no cross‐amplification with the wide set of nontarget taxa occurred. Occasionally, additional PCR products, which were clearly distinguishable from the target bands, were observed (see Appendix S2, Supporting information). The assays also proved to be highly sensitive: in the presence of cormorant DNA, 25 or less double‐stranded DNA template molecules (Table 1) were sufficient to generate amplicons with a signal strength above 0.07 RFU in the qiaxcel system. The *in silico* PCRs showed that of 7585 16S sequences, none produced an amplicon with any of the multiplex PCRs. Of the 59 202 COI sequences, 102 in theory produced an amplicon (Table S4, Supporting information). All of these sequences originate from samples collected in Canada, the United States or Australia, and in case the sequence is assigned to a species, its distribution does not include Europe.

### Detecting fish prey in otter spraints

**Figure 3:**
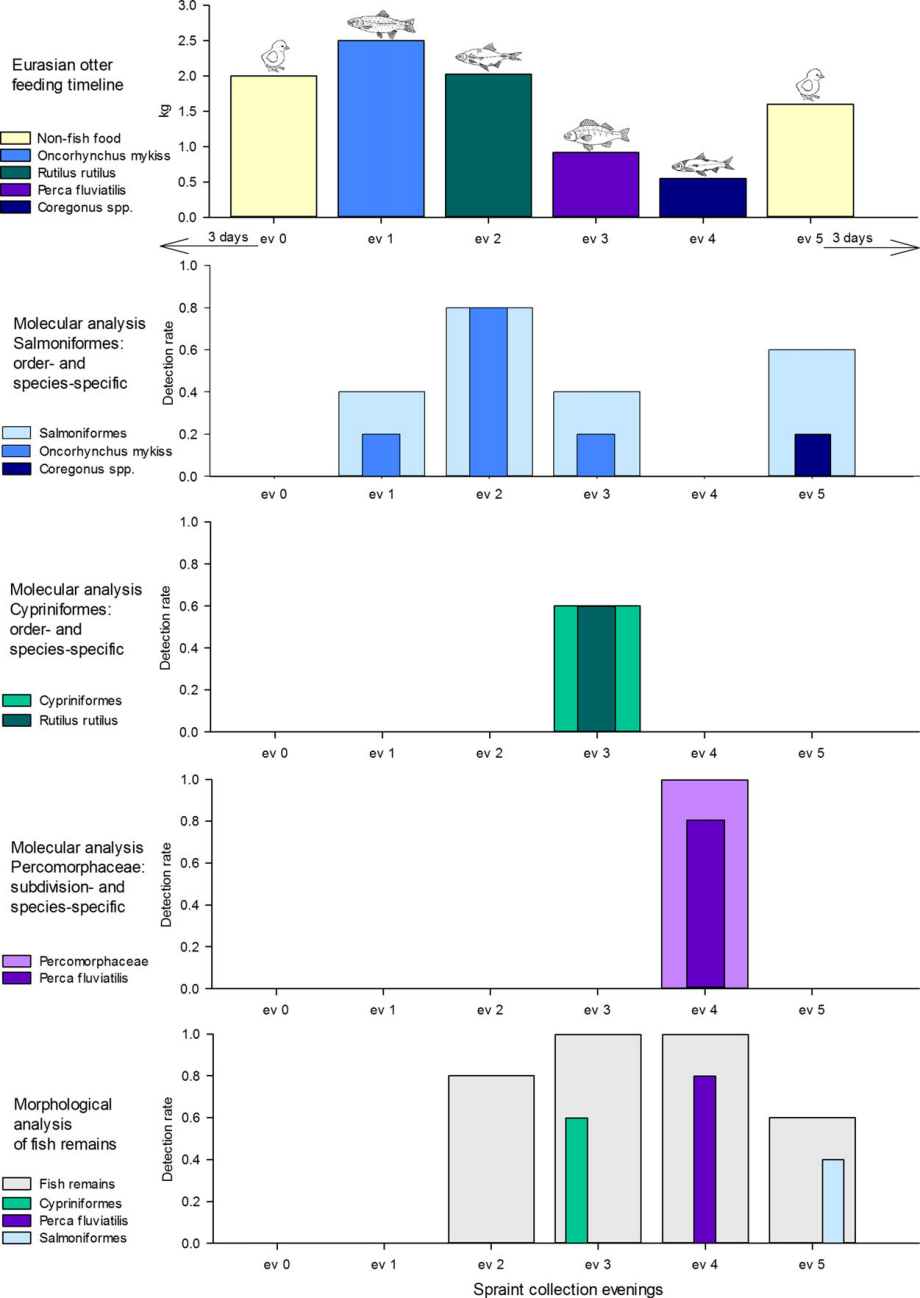
Molecular and morphological identification of fish prey in spraints of the Eurasian otter within a feeding trial. Top panel: *X*‐axis shows the order of the different prey species fed; *Y*‐axis provides the total mass of the prey items. Lower panels: *X*‐axis indicates spraint collection during evenings; *Y*‐axis displays the detection rate of fish prey (molecular or morphological). Note that molecular detections of Salmoniformes in samples collected on evenings six (0.2) and seven (0.4) are not shown.

The multiplex PCR system allowed detecting DNA from the consumed fish species 1 day after the respective feeding event: of the five spraint samples collected per evening, DNA of rainbow trout, roach, perch and whitefish was detected in four, three, four and one spraint, respectively (Fig. 3). Rainbow trout was also detected in one spraint each, collected prior and 2 days after the rainbow trout meal (Fig. 3). Moreover, DNA of Salmoniformes was detected in altogether seven spraints collected on evenings one, three, five, six and seven. Sequencing of these PCR products showed that of the seven spraints testing positive for Salmoniformes (Fig. 3), the two samples collected on evening five contained DNA of whitefish, whilst all others contained DNA of rainbow trout. The morphological analysis of the otter spraints enabled species‐specific perch identification, whereas roach and whitefish remains could be assigned to family/order level only; nonidentifiable fish remains were found in another eight spraint samples (Fig. 3). Overall, species‐specific prey detection was significantly higher for the molecular (65%) compared to morphological analysis (20%; χ^2^ = 4.14; *P* = 0.042). All samples testing negative with the FishTax assay produced an amplicon in the spike PCR.

### Detecting fish DNA in field‐collected dietary samples

**Figure 4:**
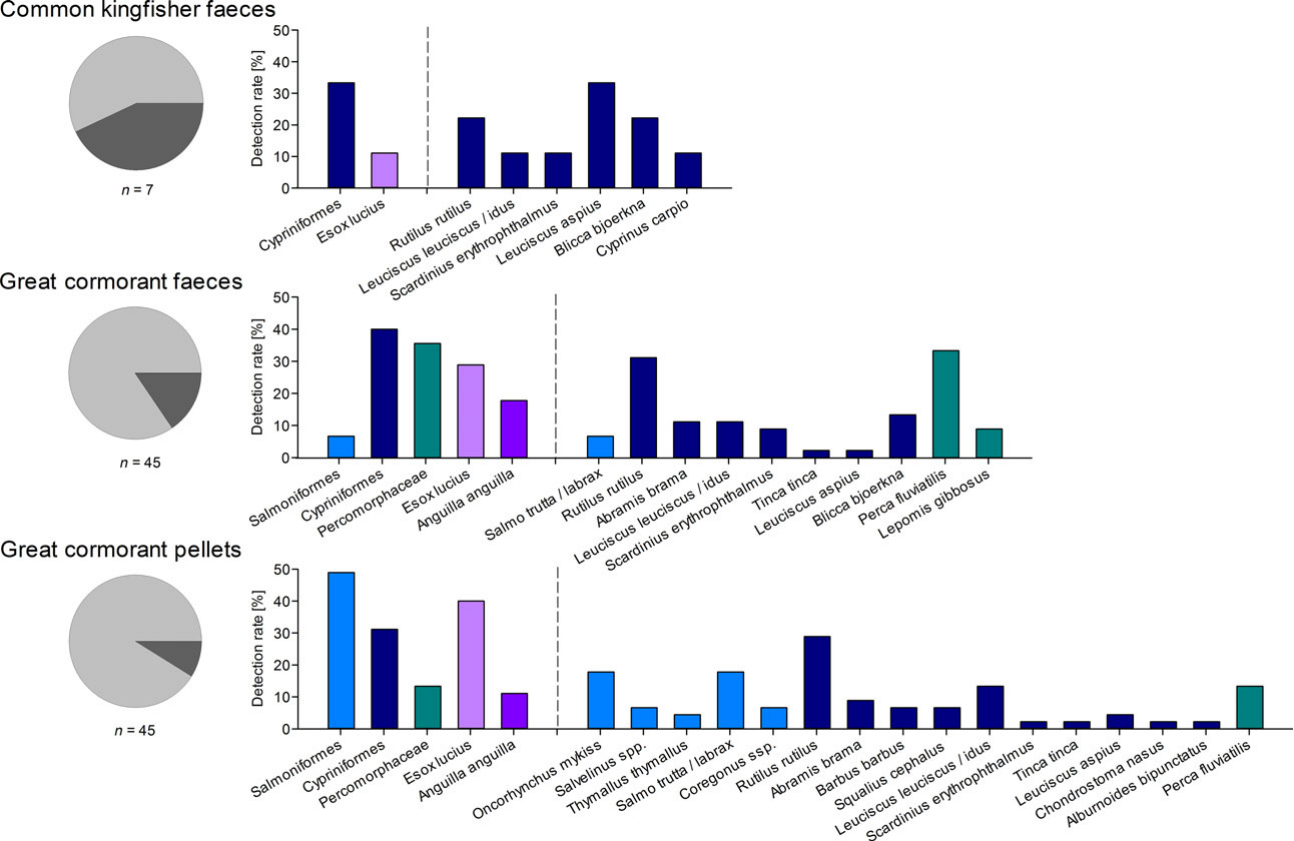
Fish DNA detected in field‐collected faeces (Common Kingfisher, Great Cormorant) and pellets (Great Cormorant) via the multiplex PCR system. Pie charts display the percentage of positive (light grey) and negative (dark grey) samples with amplifiable fish DNA, whilst bar charts show detection rates (%) per target taxon within the FishTax and the follow‐up assays left and right of the dotted line, respectively. Note that species‐specific bars do not add up to the detection rate at family level as one sample can test positive for more than one species.

The six multiplex PCR assays enabled the detection of semidigested fish DNA in field‐collected dietary samples of kingfishers and cormorants. Of seven kingfisher faeces, four yielded amplicons in the FishTax assay including one and three samples positive for pike and Cypriniformes, respectively. When applying the three CypForm assays to the latter samples, six species were identified with asp (*Leuciscus aspius*) being present in all of them (Fig. 4).

Testing 45 cormorant faecal samples with the FishTax assay resulted in 84% of samples positive for at least one target taxon; Cypriniformes were the most frequently detected group. Additionally, DNA of pike, European eel, Salmoniformes and Percomorphaceae was amplified. Subjecting the respective samples to the CypForm 1–3, PercMorph and SalForm assays resulted in the identification of seven cyprinid taxa, perch and common sunfish (*Lepomis gibbosus*), and *S. trutta*/*S. labrax*, respectively (Fig. 4). Of 45 cormorant pellet samples, 91% tested positive for fish DNA in the FishTax assay, and detections were allocated to the same five target groups as found in cormorant faeces. Regarding pellet extracts, Salmoniformes were most frequently detected including five different genera/species (Fig. 4). Whilst only perch was amplified within the PercMorph assay, ten different cyprinid species were detected through assays CypForm 1–3 (Fig. 4).

To verify the identification of fish prey by diagnostic PCR, up to five PCR products per detected fish genus or species were sequenced. In all cases, the obtained sequences matched the targeted taxon. With the exception of one kingfisher faeces, all samples testing negative with the FishTax assay produced an amplicon in the spike PCR.

## Discussion

### Multiplex PCR system

The two‐step multiplex PCR approach presented here provides an alternative to work‐intensive and expensive sequence‐based methods of prey identification. It is ideal for situations in which a defined set of prey taxa needs to be examined within a large number of individual dietary samples. The two‐step system, where prey is first identified in PCR at a high taxonomic level followed by PCRs for species‐specific identification within the respective taxon, permits cost‐efficient screening for a large number of taxa. This extends the application of diagnostic multiplex PCR to research in environments where higher numbers of prey species need to be examined such as studies on the diet of piscivores in Central European freshwaters.

Our multiplex PCR system encompasses 78 Central European fish and lamprey species and enables the detection and identification of 31 species, six genera, two families, two orders and two fish family clusters. The detection system focuses on characteristic lotic and lentic species of the Alpine foreland, their companion species, and fish being relevant for commercial and recreational fishing according to the Water Framework Directive of the European Union (European Parliament & Council of the European Union 2000). Fish species which were targeted only by group‐specific primers in the FishTax assay and red‐listed fish species which were not considered in this study (Appendix S1, Supporting information) are either endangered, invasive or are small bottom‐dwelling fish occurring in large rivers such as Danube or Rhine. According to their limited distribution, these species are unlikely to constitute a frequently used prey. NGS techniques, already implemented for dietary analyses of marine piscivores such as penguins (Jarman *et al*. 2013) or seals (Deagle *et al*. 2009), have the potential to detect DNA of these species (Glenn 2011; Pompanon *et al*. 2012; Shokralla *et al*. 2012). In case consumption of rare species needs to be assessed, such NGS techniques can complement the presented diagnostic system. The multiplex PCR approach, even when applied in a stepwise manner, will be stretched to its limits when an even greater variety of fish species need to be identified in dietary samples. Under such circumstances, NGS technologies, which are rapidly evolving with decreasing costs per sample (Glenn 2014), will provide a valuable alternative. These sequence‐based approaches of prey DNA identification will also benefit from the new sequences generated in this study.

Our diagnostic system, nevertheless, is broadly applicable to assess the feeding ecology of fish‐eating invertebrates and vertebrates in Central Europe. It has the potential to be used in other regions such as northern and western Europe too, as several of the presented primers should also work with species outside the Central European range. For example, the genus‐specific primer pair targeting whitefish is based on three whitefish species occurring in the Alpine foreland of Austria and Germany. Yet all online available COI sequences of 24 whitefish species match well with the designed primers (forward and reverse primers have one and two mismatches maximum, respectively). Correspondingly, other primers presented here might be suitable for use outside the study area. Testing their specificity against herein not considered nontarget taxa, which are native to the respective study area, is strongly recommended to avoid false‐positive amplifications. This includes, as outlined in this study, sequencing of PCR products generated form field‐collected samples to confirm amplicon identity. Furthermore, primers which produced an amplicon during the *in silico* PCRs should not be applied in North America/Australia without further specificity tests. The application of the six assays will also not be restricted to dietary samples of the three species examined here; they will be technically working with at least seven other Central European piscivores whose DNA was included in our nontarget testing. Amongst these are five bird species, the northern raccoon (*Procyon lotor*), currently invading Central Europe (Michler *et al*. 2009) and the grass snake (*Natrix natrix*).

The sensitivity of the multiplex PCR assays was high across the board, enabling successful amplification based on as little as 25 template molecules. The high sensitivity combined with the balanced primer concentrations, ensuring similar amplification efficacies for each target taxon, safeguards against detection dropouts caused by differing amounts of prey species’ DNA in dietary samples (Sint *et al*. 2012). Moreover, the high assay sensitivity should counteract the lower detection probability of longer prey DNA fragments which are usually present in minute quantities (Deagle *et al*. 2006).

The multiplex PCR system presented here provides also a straightforward approach in terms of its practical implementation: once the DNA has been extracted from the samples, these can be analysed quickly and at comparably low cost. For example, it took one person 2 days to subject the 90 cormorant samples to the FishTax assay and the second‐step PCRs, to run the electrophoresis and to tabulate the screening results. The average screening costs per sample for consumables was about € 3.5, and all work can be performed with basic molecular laboratory equipment.

### Otter feeding trial and spraint analysis

In the otter feeding trial, the molecular detection system outperformed the morphological analysis of prey remains with regard to species‐specific prey identification. However, only on evenings two and four the molecular assays reached a prey detection rate of 80% (rainbow trout (*Oncorhynchus mykiss*) and perch (*Perca fluviatilis*), respectively) in the spraints. The question arises, as why the highly sensitive assays could not detect the respective fish DNA in all of the samples. Amplicon size seems to be negligible in this regard, because the primer pair producing the longest fragment (Percomorphaceae; 375–383 bp) scored the highest detection rate. PCR inhibitors, which could get copurified during DNA extraction of the spraint samples, were also ruled out as amplification was not blocked in the spike PCR. Other factors which could explain our findings include differences in protein and lipid content of the fed fish species, different meal sizes and the occurrence of empty spraints. A high lipid content in prey fish seems to reduce mitochondrial DNA degradation through digestion as shown by Thomas *et al*. (2014) in a feeding trial on harbour seals. This observation fits to the presented findings as Schreckenbach *et al*. (2001) found higher crude fat proportions in rainbow trout (11.57%) compared to roach (1.94%) and whitefish (6.39%). The small whitefish meal (552 g, 4 fish) could explain the low detection rates of this prey, because it is likely that not all otters had a share of this meal. Finally, in a feeding trial on captive otters, Carss & Parkinson (1994) found approximately one‐third of the collected otter spraints (*n* = 1544) to be anal jelly secretions not containing any morphologically identifiable fish remains. In the presented trial, three spraints, one from evening two and two from evening five, neither contained morphologically identifiable fish remains nor was fish DNA detected. Presumably, these spraints were anal jelly secretions and such samples should be excluded in future dietary studies based on otter spraints.

DNA of Salmoniformes, precisely rainbow trout, was detected in some spraints collected before and more than 1 day after the otters were fed this species. Whilst the detection event 2 days postfeeding could be attributed to differences in gut passage times between otters, environmental DNA contamination is a likely explanation for the other detections as rainbow trout constitutes a major part of the otters’ usual diet at the Alpenzoo. Furthermore, otters sometimes hide their prey (Ruiz‐Olmo 1995), thus remains of previous meals could have contributed to the contamination of spraints with environmental DNA.

### Field‐collected dietary samples

Fish species such as pike, roach and bleak, detected in the kingfisher faeces by the two‐step multiplex PCR system, have been previously identified as part of this bird's diet in Central Europe (Čech & Čech 2011) and strengthen the species’ image as a generalist fish eater (Vilches *et al*. 2013). The fish were most likely juveniles, caught in still waters in the alluvial forests along the main riverbeds, as the maximum prey size of the kingfisher is 100 mm fork length (Cramp 1985). The seven faecal samples were contaminated with soil when they were collected; as soil material is known to cause problems with molecular prey detection in bird droppings (Oehm *et al*. 2011), this is a likely explanation for the negative result in the spike PCR. For future studies, an optimization of the DNA extraction protocol could prevent negative results caused by inhibition (Zarzoso‐Lacoste *et al*. 2013). Additionally, performing the multiplex PCRs more than once on a subset of samples could help to determine the robustness of the approach for field‐collected samples.

The application of the six multiplex PCR assays to field‐collected cormorant faeces and pellets lead to the detection of 12 and 18 fish species/genera, respectively. This reflects the broad prey spectrum cormorants utilize in Alpine foreland freshwaters which are characterised by a diverse fish fauna (Marzano *et al*. 2013). Previous studies applying morphological prey identification to pellets have struggled to identify cyprinid species as their hard parts are usually not species‐specifically distinguishable (Keller 1998). This problem is remedied by our DNA‐based approach that identified nine cyprinid species and one genus in the 45 tested pellets. In 9% of the pellets, no fish DNA could be detected. This does not come as a surprise as cormorants are known to produce empty pellets as juveniles, at food shortage, or under stress (Zijlstra & Vaneerden 1995). Likewise, the cormorant faeces wherein no fish DNA was detected (16%) most likely contained urea as main component and hardly any prey DNA.

### Conclusion and outlook

The two‐step multiplex PCR approach presented here provides an efficient, easy to use and cost‐effective tool to examine the diet of piscivores in great detail. Although the system has been developed for Central Europe, it will be applicable to other regions where the targeted fish species occur; however, we strongly recommend evaluating specificity *a priori*. Furthermore, the application of the multiplex PCR system is not restricted to prey identification, but the assays or single primer pairs will be useful to any approach where fish DNA needs to be identified with high specificity and sensitivity such as environmental monitoring, studies on environmental DNA (Rees *et al*. 2014) or species‐specific identification of fish eggs, larvae and carcasses (Hubert *et al*. 2015). Finally, the use of blocking primers (Vestheim *et al*. 2011) in combination with the here presented multiplex PCR assays could promote dietary studies on fish themselves.

B.T., J.O. and M.T. conceived and designed the study. J.O. generated the reference sample collection with the help of B.T. B.T. designed the primers, established the molecular assays and performed laboratory work together with H.M., A.O. and C.Z. B.T. and A.O. carried out the feeding trial; J.O. and A.O. carried out the morphological analysis. B.T. analysed the data, compiled tables and figures and wrote the manuscript which was revised and improved by J.O. and M.T.

## Data accessibility

DNA sequences: GenBank Accession nos KR476824–KR476986 for 16S and KR476987–KR477299 for COI sequences. The screening results of the otter feeding trial and of the field‐collected samples as well as 16S and COI alignments of all sequences submitted to GenBank have been archived in the Dryad Digital Repository (doi:10.5061/dryad.1vf74).

## Supporting information


**Table S1.** The taxonomy of all Central European fish species targeted by the multiplex PCR assays including the number of DNA extracts generated from tissue samples and their origin.

**Appendix S1.** Red list freshwater fish species of Central Europe not included in this study.

**Table S2.** Non‐target animals used to evaluate the specificity of the multiplex PCR assays including the number of DNA extracts tested per taxon.

**Appendix S2.** Notes on the multiplex PCR assays.

**Table S3.** Collection locations of the Common Kingfisher (*Alcedo atthis*) a faeces.

**Table S4.** *In silico* PCR conditions and results.
