# Supplementary material for: Molecular prey identification in Central European piscivores
Source: Mol Ecol Resour. 2015 Jun 21;16(1):123–37. doi: 10.1111/1755-0998.12436 (PMC4744964; doi:10.1111/1755-0998.12436)
Supplement: Supplementary file 2 — Appendix S1. Red list freshwater fish species of Central Europe not included in this study. [file MEN-16-123-s002.docx]

**Supplementary Information 2**

**Red list freshwater fish species of Central Europe not included in this study**

Red list species Austria ([Wolfram & Mikschi 2007](#_ENREF_2)): *Ballerus ballerus*, *Umbra krameri*, *Sander volgensis*, *Vimba elongata*, *Babka gymnotrachelus*.

Red list species Germany ([Freyhof 2009](#_ENREF_1)): *Alosa alosa, Ameirus melas, Cottus* spp.*, Eudontomyzon vladykovi, Lampetra fluviatilis, Misgurnus anguillicaudatus, Neogobius fluviatilis, Osmerus eperlanus,* deep lake *Salvelinus* spp., *Umbra pygmaea.*

**References**

Freyhof J (2009) *Rote Liste der im Süßwasser reproduzierenden Neunaugen und Fische (Cyclostomata & Pisces). Fünfte Fassung.* Naturschutz und Biologische Vielfalt (Bundesamt für Naturschutz).

Wolfram G, Mikschi E (2007) *Rote Liste der Fische (Pisces) Österreichs.* Böhlau-Verlag, Wien, Köln, Weimar.
