# Supplementary material for: Molecular prey identification in Central European piscivores
Source: Mol Ecol Resour. 2015 Jun 21;16(1):123–37. doi: 10.1111/1755-0998.12436 (PMC4744964; doi:10.1111/1755-0998.12436)
Supplement: Supplementary file 4 — Appendix S2. Notes on the multiplex PCR assays. [file MEN-16-123-s004.docx]

**Supplementary Information 4**

**Notes on the multiplex PCR assays**

In case of high concentrations of target DNA and/or when DNA of a not targeted fish species was present, some of the multiplex PCR assays produced additional amplicons. All of these were easily differentiable from the expected fragment sizes and did not interfere with target identification. FishTax and CypForm 2 multiplex PCRs did not display any of these peculiarities, whereas in the SalForm assay an additional amplicon appeared at ~375 bp when testing the mixture of target DNA molecules at a concentration equal or higher than 200 DNA double strands (further on “ds”) per target species in PCR; this could not be observed in field-collected samples. Likewise, the target DNA mixture of the PercMorph assay produced one additional amplicon at ~150 bp and another one at ~325 bp. The CypForm 1 assay produced two additional amplicons (~140 bp, ~160 bp) in case DNA of *Rutilus rutilus* and *Alburnus mento* was present in a sample at concentrations >200 DNA ds in PCR; these non-target bands were observed in both positive controls and field-collected samples. In the CypForm 3 assay the amplicon sizes of *Chondrostoma nasus* and *Blicca bjoerkna* differ only by 8 bp, demanding a good resolving power of the electrophoretic system. The CypForm 3 assay also produces a ~446 bp band with the DNA of *Leuciscus leuciscus* even though this species is not specifically targeted in this reaction. The band is produced by the primers Bli-bjo-S675 and Vim-vim-A677 and sequencing confirmed it to be the COI sequence of *Leuciscus leuciscus*. The closely related *Leuciscus idus* has an additional mismatch at the 3’ end of the Vim-vim-A677 primer and is thus not amplified by this primer combination. Bli-bjo-S675 and Vim-vim-A677 could theoretically be used to distinguish *Leuciscus leuciscus* from all other fish species in the multiplex PCR system. However, we’d like to point out that both primers do not fit perfectly onto the DNA of this species (one, respectively two mismatches) and the produced fragment is rather long. Thus it does not meet the standards of the presented multiplex PCR system and we refrained from including it.
